# TNF-α inhibitor tanfanercept (HBM9036) improves signs and symptoms of dry eye in a phase 2 trial in the controlled adverse environment in China

**DOI:** 10.1007/s10792-022-02245-1

**Published:** 2022-02-22

**Authors:** Yanling Dong, Shuang Wang, Lin Cong, Ting Zhang, Jun Cheng, Nannan Yang, Xiaohong Qu, Dongfang Li, Xueying Zhou, Holly Wang, Michael Lee, Meng Wang, Stephen Chen, George W. Ousler, Xiaoxiang Chen, Lixin Xie

**Affiliations:** 1 Qingdao Eye Hospital of Shandong First Medical University, State Key Laboratory Cultivation Base, Shandong Provincial Key Laboratory of Ophthalmology, Eye Institute of Shandong First Medical University, Qingdao, China; 2 Harbour BioMed, Shanghai, China; 3 Ora Inc, Andover, MA USA

**Keywords:** Dry eye disease, TNF-TNFR1 inhibitor, Tanfanercept, Controlled adverse environment

## Abstract

**Purpose:**

This study evaluated the clinical safety and efficacy of tanfanercept (HBM9036) ophthalmic solution as a novel treatment for dry eye disease (DED) in a controlled adverse environment (CAE) study conducted in China.

**Methods:**

In a single-center, double-masked, randomized, placebo-controlled study, 100 patients received 0.25% tanfanercept, or placebo, twice daily for eight weeks. A mobile international CAE^®^ DE Model was used for patient selection with a standardized challenge endpoint. Primary efficacy endpoint was fluorescein inferior corneal staining score (ICSS) pre- to post-CAE challenge from baseline. Secondary endpoints included Schirmer’s Tear Test, Tear-Film Break-Up Time, Ocular Discomfort Score, Ora Calibra^®^ Ocular Discomfort and 4-Symptom Questionnaire, total corneal staining score (TCSS), and drop comfort. Signs and symptoms were assessed both pre- and post-CAE to evaluate the efficacy of tanfanercept on both environmental and CAE endpoints.

**Results:**

The tanfanercept treatment group showed improvement in ICSS pre- to post-CAE change from baseline scores when compared to placebo (− 0.61 ± 0.11 and − 0.54 ± 0.11, respectively; mean difference = 0.07, *p* = 0.65). TCSS pre–post-CAE change from baseline scores was also in favor of active when compared to placebo (− 1.03 ± 0.21 and − 0.67 ± 0.21, respectively; mean difference = 0.37, *p* = 0.23). Schirmer’s score improvement was demonstrated in favor of active (1.87 ± 0.62 mm) as compared to placebo (1.28 ± 0.62 mm; mean difference = 0.59 mm, *p* = 0.50). Change from baseline in mean Tear-Film Break-up Time favored active treatment over placebo (mean difference = 1.21 s, *p* = 0.45). Notably, the tanfanercept showed more obvious benefits for each DED sign in a subgroup of subjects ≥ 35 years of age. Tanfanercept was well tolerated with no serious adverse events occurring during the study.

**Conclusion:**

Tanfanercept demonstrated improvements in favor of active as compared to placebo in the signs of DED, being safe and well tolerated. These data support further evaluation of tanfanercept for the treatment of DED in China.

**Trial registration:**

This study was retrospectively registered at ClinicalTrials.gov (NCT04092907) on September 17, 2019.

## Introduction

Dry eye disease (DED) is a complex chronic disease that results in symptoms of discomfort, visual disturbance, and tear film instability. In China, as much as 31% of the population aged 5–89 experience symptoms of DED (approximately 371 million people) [1]. Similarly in the USA, as many as 3.2 million women and 1.7 million men over the age of 50 have DED, with a projected 40% increase in the number of patients affected by 2030 [2–4]. DED represents considerable public health burden faced in China and across the world. Currently, the primary therapeutic strategy for the management of DED aims to improve dry eye symptom through instillation of artificial tears to provide lubrication and supplementation of the patient’s natural tears. Targeting of DED symptoms through artificial tears provides patients symptom relief but does not modulate the underlying DED signs or disease progression. With the aging population in China and other countries, increasing computer and smartphone use, and high prevalence of fine dust and environmental pollutants, DED is expected to become more prevalent, and the development of safe, tolerable, and broadly effective treatments against both the signs and symptoms of DED is becoming more important [5].

As an alternative therapeutic strategy to tear supplementation, signs and symptoms of DED can be improved by treating the underlying cause of the disease: inflammation. Currently, Restasis^®^ (cyclosporine ophthalmic emulsion), Cequa^®^ (nanomicellar solution of cyclosporine), and Xiidra^®^ (lifitegrast ophthalmic solution) which target ocular surface inflammation have been approved in the USA for the treatment of DED. These treatments address a sign and/or symptom of DED. However, currently there is only one approved anti-inflammation drugs for DED in China (0.05% cyclosporine ophthalmic emulsion). Also only small proportions of patients respond to treatment with Restasis^®^ after a relatively long period of treatment (15% of patients after 6 months of treatment) and all three therapies have many reports of ocular adverse events and burning and stinging upon drop instillation [6–8]. There remains a need for improved treatment options, especially for Chinese patients.

Toward the development of alternative treatment options for DED targeting the underlying inflammation of DED, tanfanercept, also known as HBM9036 and HL036 (HanAll BioPharma Co., Ltd., Seoul, South Korea) Ophthalmic Solution, is a molecularly engineered TNF receptor 1 (TNFR1) fragment formulated as an eye drop. Tumor necrosis factor (TNF) has been identified as a major cytokine mediating the inflammatory component of various diseases including DED [9–11]. Strong binding of soluble TNF to two receptors TNFR1 (p55) and TNFR2 (p75) results in the induction of inflammation and pathogenesis of disease [12]. As such, inhibitors of TNF-TNFR binding are currently in development and have been approved for diseases including rheumatoid arthritis, psoriasis, ankylosing spondylitis, ulcerative colitis, uveitis, and Crohn’s diseases [13]. Biologic, antibody-based therapeutics have proven most effective in reducing TNF-mediated inflammation; however, the large molecular size (~ 150 kDa) limits bioavailability and ocular tissue penetration [14]. Due to these limitations, there is a need for topical eye drop forms of TNF-inhibitors with increased penetration and ocular distribution and minimal systemic side effects.

Tanfanercept eye drops demonstrated potent anti-inflammatory effects in a carrageenan-induced acute in vivo model of inflammation and significant efficacy in a collagen-induced arthritis model. HBM9036 Ophthalmic Solution has also been shown to cause significant clinical improvements in several DED animal models [15] and has demonstrated safety from a phase 1 study conducted in healthy volunteers. In a multi-center, phase 2, randomized, double-masked, placebo-controlled, parallel-arm study conducted in the USA (VELOS-1, NCT03334539), tanfanercept treatment demonstrated statistically significant improvements in the signs, specifically reduction of inferior corneal staining, and symptoms, notably ocular burning, of DED compared to placebo treatment.

The objective of this study is to evaluate the clinical safety and efficacy of 0.25% tanfanercept ophthalmic solution as a novel treatment for DED in Chinese adult patients with moderate-to-severe DED.

## Methods

### Study design

**Fig. 1:**
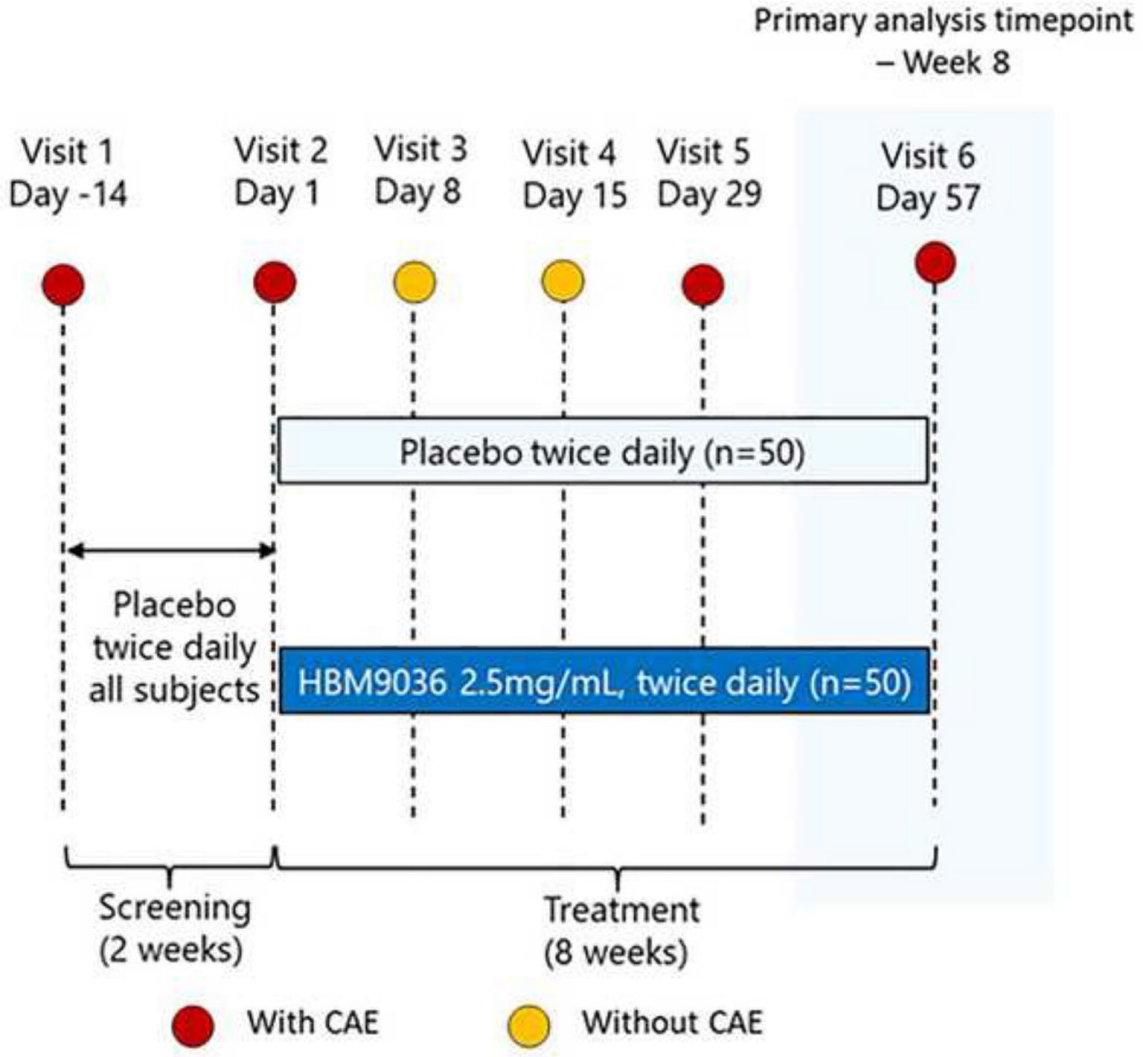
Clinical study plan. Subjects were screened for inclusion criteria at Visit 1. During the screening period encompassing Visits 1 and 2, two 90-min exposures to the mobile, international Ora CAE^®^ Dry Eye Model were conducted to determine eligibility to enter into the treatment period of the study. Qualifying subjects who demonstrated potential response to CAE challenge in DED sign and symptom were randomized in a double-masked fashion into one of two treatment arms: 0.25% tanfanercept treatment or placebo treatment. All subjects were instructed to self-administer treatment (active or placebo) BID. At Visits 3 and 4, subjects were not exposed to the CAE but DED signs and symptoms were assessed. At Visits 5 and 6, subjects faced CAE challenge, while DED signs and symptoms were assessed pre-, during (symptom assessments only), and post-challenge

This was a single-center, randomized, double-masked, placebo-controlled study conducted in China at Qingdao Eye Hospital (100 subjects) with enrollment between March 22, 2019, and July 10, 2019. Treatment arm assignment was masked to subjects, sponsor, contract research organization, and site personnel. One hundred patients were randomized 1:1 to receive tanfanercept (HBM9036) or placebo. Subjects received treatment as assigned twice daily over two study periods, screening and treatment, spanning six visits over a duration of eight weeks. The study design and timeline of assessments is detailed in Fig. 1. During the screening period, consisting of Visits 1 and 2, subjects were exposed to the Ora CAE^®^ Dry Eye Model. The CAE is an established model that standardizes environmental conditions by regulating humidity, temperature, airflow, lighting conditions, and visual tasking [16, 17]. Dry eye patients are exposed to all of these conditions simultaneously for 90 min and undergo clinical evaluations before and after CAE exposure, as well as symptom assessments during the challenge. The CAE represents everyday situations that dry eye patients encounter (e.g., forced air heating systems, airplane travel, computer use) and allows for the standardization of these influential factors. Standardizing these factors enables a controlled assessment of interventions intended to treat the signs and/or symptoms of dry eye. In order to qualify for randomization and continue to the treatment period, a subject must have demonstrated a positive response to CAE exposure at Visit 1 and a similar response in the same eye at Visit 2 (Day 1).

Qualified subjects from the screening phase were randomized and continued to the treatment period of the study. During this period, the subjects received tanfanercept or placebo in a double-masked fashion for 56 days for self-administration twice daily (BID) in the morning and evening. At Visit 3 (Day 8) and 4 (Day 15), subjects were not challenged with CAE exposure, but sign and symptom assessments were conducted. At Visits 5 (Day 29) and 6 (Day 57), subjects were exposed to the CAE, with assessments of DED sign and symptom pre-CAE, during CAE (ocular discomfort self-assessments only), and post-CAE exposure.

Signs of DED were assessed by fluorescein corneal staining of all ocular regions (central, superior, inferior, temporal, nasal) measured by the Ora Calibra^®^ Corneal and Conjunctival Fluorescein Staining Scale (Ora Calibra, Andover, MA, USA) ranging from 0 = none to 4 = severe. For total corneal sum staining (TCSS), which is a summation of staining scores across all corneal regions, scores range from 0 to 12. Fluorescein corneal staining was assessed at Visits 1, 2, 4, 5, and 6. Tear-film Break-Up Time (TFBUT) in seconds was assessed at all visits. Conjunctival redness, as assessed by the Ora Calibra^®^ Conjunctival Redness Scale ranging from 0 = none to 4 = severe, was measured at all visits. Unanesthetized Schirmer’s test was assessed in millimeters at Visits 1, 2, 4, 5, and 6 post-CAE challenge only at visits where CAE challenge was conducted. Since the examination results of fluorescein corneal staining and Schirmer’s test in visit 3 (day 8) are relatively stable or have little changes compared to visit 2 (day 1), and in order to lighten the burden on patients and optimize study procedure, in visit 3, these two tests were not performed.

Symptoms of DED were measured throughout the study at each office visit. The secondary analysis of the change from baseline (Visit 2) in Ora Calibra^®^ Ocular Discomfort and 4-Symptom Questionnaire for dry eye (burning, dryness, grittiness, and stinging) (Ora Calibra, Andover, MA, USA) from to Visit 6 (Day 59) as assessed by the Ora Calibra^®^ Ocular Discomfort Scale was used to determine if treatment with 0.25% tanfanercept was providing clinically important improvements in the dye eye symptoms experienced by subjects. Dry eye symptoms were measured using the Ora Calibra^®^ Ocular Discomfort Scale ranging from 0 = none to 4 = severe, the Ora Calibra^®^ Ocular Discomfort and 4 Symptom Questionnaire with scores ranging from 0 = none to 5 = most, and Visual Analog Scale (VAS) with scores ranging from 0% = no discomfort to 100% = maximal discomfort at all visits [18]. Symptoms and visual tasks assessed by the Ocular Surface Disease Index© (OSDI) Questionnaire (scores ranging from 4 = “all of the time” to 0 = “none of the time” for 12 questions) were measured at all visits [19]. OSDI assessments were conducted, before CAE exposure only, at Visits 1, 2, 5, and 6.

This study was conducted in compliance with current Good Clinical Practices, including the International Conference on Harmonisation Guidelines and the Declaration of Helsinki. In addition, informed consent was obtained from each subject before any study-specific procedures were performed. This study was registered at ClinicalTrials.gov (NCT04092907).

### Screening and study entry criteria

Individuals at least 18 years of age were eligible to participate in this study if they had provided written consent, been willing and able to comply with all study procedures and had a self-reported history of DED for at least six months prior to enrollment. Additionally, the individuals must have had a history of use or desire to use eye drops for management of DED symptoms within 6 months of Visit 1. The following baseline ocular characteristics were required for all subjects to enroll in the study: a best-corrected visual acuity (BCVA) of 0.7 logMAR or better in each eye at Visit 1, a score of ≥ 2 for at least one of the DED symptoms according to the Ora Calibra^®^ Ocular Discomfort and 4-Symptom Questionnaire at Visits 1 and 2, a score of ≤ 10 mm and ≥ 1 mm at Visits 1 and Visits 2 for the Schirmer’s test, a staining score in at least one corneal region ≥ 2 at Visits 1 and 2 according to the Ora Calibra^®^ Corneal and Conjunctival Fluorescein Staining Scale, and a redness score ≥ 1 at Visits 1 and 2.

Subjects were excluded from the study if they had any clinically significant slit-lamp findings at Visit 1 including active blepharitis, meibomian gland dysfunction, lid margin inflammation, active ocular allergies requiring therapeutic treatment, and/or that which in the opinion of the investigator that might interfere with the study parameters. In addition, subjects diagnosed with an ongoing ocular infection, or active ocular inflammation at Visit 1 were not allowed to enroll. Subjects having previously had laser-assisted in situ keratomileusis (LASIK) surgery or had a femtosecond small incision lenticule extraction (SMILE) within the last 12 months, had phacoemulsification within the last 3 months, or had dry eye or aggravation of dry eye caused by other ocular operations were excluded from the study. Lastly, subjects were excluded from the study if they had used ophthalmic cyclosporine A, tacrolimus, or Xiidra^®^ within 60 days prior to Visit 1.

Subjects who met all of the above inclusion criteria and none of the exclusion criteria underwent exposure to the CAE during Visits 1 and 2 to screen for exacerbated responses in sign and symptom of DED. The screening period allowed for selection of subjects who had demonstrated a potential to respond to both signs and symptoms of DED and were continued into the treatment period of the study. An exacerbated CAE response was defined as ≥ 1 increase of post-CAE (i.e., pre- to post-change) inferior corneal staining score (ICSS) in the study eye, an Ocular Discomfort Score (ODS) ≥ 3 in at least two consecutive measurements in the study eye during CAE, and an ODS ≥ 4 in at least 2 consecutive measurements in the study eye if ODS = 3 at time (*T*) = 0.

### Efficacy and safety endpoints

The primary efficacy endpoint of the study was mean change from baseline of inferior corneal staining score (ICSS) pre- to post-CAE exposure in the designated study eye as addressed by the Ora Calibra^®^ Corneal and Conjunctival Staining Scale for Grading of Fluorescein staining at Visit 6 (Day 57, Week 8). Assessment of change from baseline in the change from pre- to post-CAE exposure allows for the standardized and controlled assessment of the efficacy of tanfanercept in alleviating exacerbations of the signs of DED.

The secondary efficacy endpoints evaluating tanfanercept against signs of DED were changes from baseline in change from pre- to post-CAE ICSS of the study eye, changes from baseline in post-CAE conjunctival redness score of the study eye, changes from baseline in Schirmer’s test (non-anesthetized), and changes from baseline in post-CAE TFBUT in the study eye. Secondary endpoints evaluating the efficacy of tanfanercept in alleviating the symptoms of DED were changes from baseline in pre-CAE Ora Calibra^®^ Ocular Discomfort and 4-Symptom Questionnaire of the study eye according to Ora Calibra^®^ Ocular Discomfort Scale, changes from baseline in pre-CAE symptom score according to Ora Calibra^®^ Ocular Discomfort and 4-symptom questionnaire, changes from baseline in OSDI© scores, changes from baseline in pre-CAE burning sensation score evaluated according to VAS, and changes from baseline in average weekly burning sensation score in a subject diary.

Safety measures including the incidence and severity of ocular and non-ocular adverse events (AEs), BCVA at all visits, findings from slit-lamp biomicroscopy were assessed at all time points. Drop comfort measured at Visit 2, as an evaluation of tolerance, was assessed by the Ora Calibra^®^ Drop Comfort Scale and the Ora Calibra^®^ Drop Comfort Questionnaire at Visit 2, intraocular pressure at Visit 1 and Visit 6, dilated fundoscopy at Visit 1 and Visit 6.

### Statistical methods

The study was to evaluate the efficacy and safety of tanfanercept. A total of 100 was planned. The sample size determination was based on practicality and efficacy estimate precision consideration.

Baseline characteristics were summarized on all randomized subjects. The primary efficacy analysis set was the per-protocol analysis set (PP), which excluded subjects with protocol deviations that potentially biased efficacy assessment, such as missing Week 8 efficacy assessment and poor compliance. Safety was assessed on the safety analysis set (Safety), which includes all subjects who received at least one dose of study treatment. All AEs were coded to Medical Dictionary for Regulatory Activities (MedDRA version 22.0) and World Health Organization (WHO) drug dictionaries, as appropriate. Only treatment-emergent AEs (TEAEs), which were AEs that occurred after the initiation of study treatment, were summarized and presented.

Baseline measures were defined as the last measure prior to the initiation of study treatment (generally at Visit 2, Day 1). If a measure was taken both pre-CAE and post-CAE, the baseline was the time point matched value at Visit 2. For changes from pre-CAE to post-CAE post-first treatment, the change from pre-CAE to post-CAE at Visit 2 was considered the baseline value.

Unless otherwise stated, the pre- to post-CAE changes in signs and symptoms of DED were calculated as post-CAE score–pre-CAE Score, where appropriate; change from baseline was calculated as visit–baseline; and finally, treatment comparisons between active and placebo were calculated as active–placebo.

For the primary efficacy analysis, ANCOVA models were used to compare the change from baseline in the pre- to post-CAE ICSS at Visit 6 (Day 57), between 0.25% tanfanercept and placebo including treatment and the change in pre- to post-CAE ICSS at baseline as covariates. Model-based least-squared (LS) mean was estimated and presented with corresponding standard error (SE).

In contrast to confirmatory studies, the study was an exploratory phase 2 study, which was not powered for any statistical testing. As a result, we opt not to present *p*-value here.

## Results

### Subject demographics and disposition

**Fig. 2:**
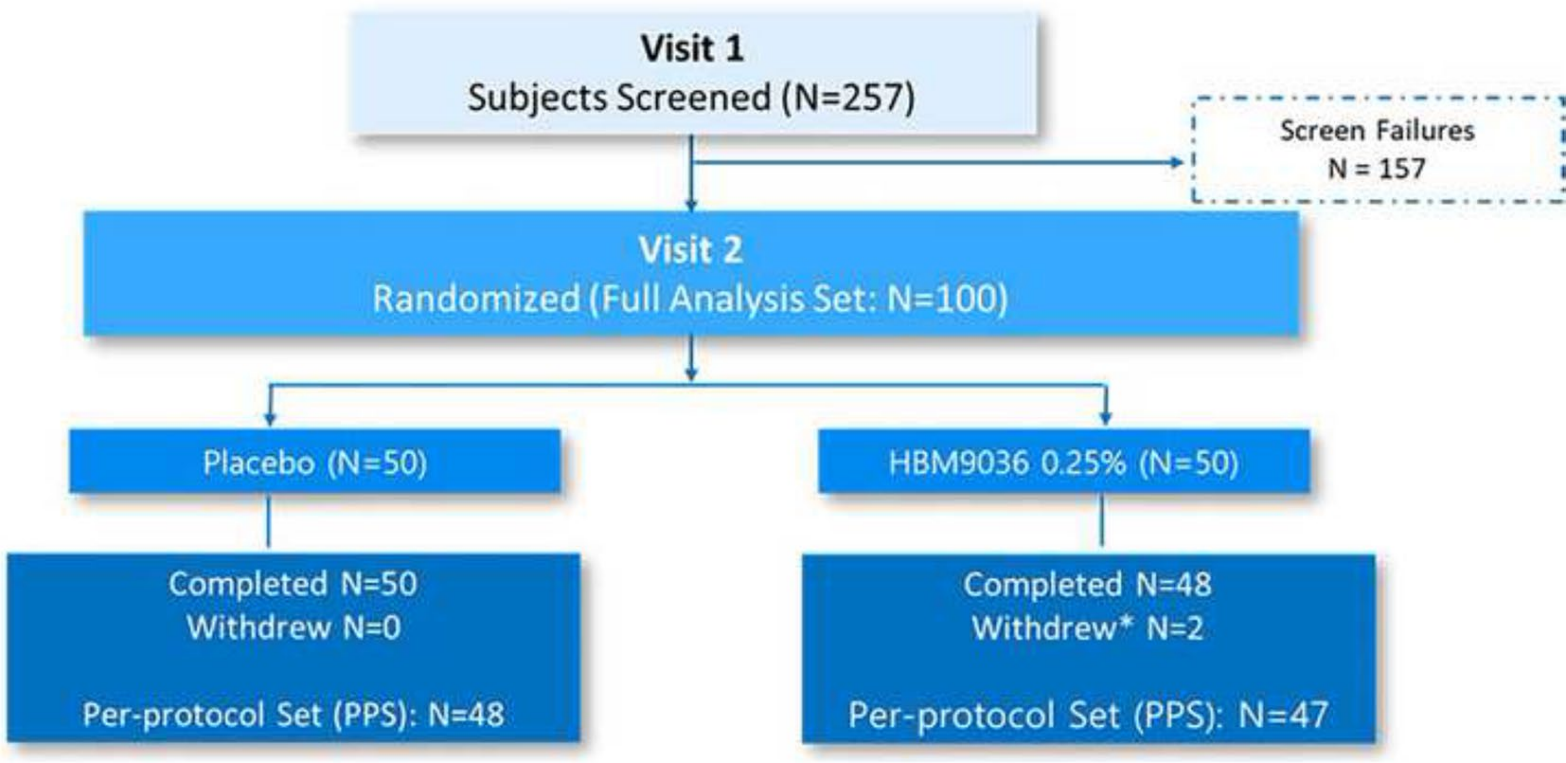
Subject disposition. In total, 257 subjects were screened at Visit 1. Following 157 screen failures, at Visit 2, 100 subjects were enrolled and randomized in a 1:1 ratio into two treatment arms, 0.25% tanfanercept (active) and placebo, resulting in 50 subjects assigned to each group. During the course of the study, two subjects in the 025% tanfanercept treatment group withdrew due to subject choice (*N* = 1) and an adverse event (*N* = 1). The 98 subjects completed the study. Due to major protocol deviations, the per-protocol analysis sets were reduced to 48 subjects in the placebo treatment group and 47 subjects in the 0.25% tanfanercept treatment group

During the screening period of the study (Visits 1 and 2), 257 subjects were screened. Of these 257 screened subjects, 100 subjects were randomized 1:1 into either 0.25% tanfanercept or placebo group. In total, 50 subjects were assigned to the 0.25% tanfanercept treatment group and 50 subjects were assigned to the placebo group. Five (5) subjects were excluded from PP: 2 due to early withdrawals (both in 0.25% tanfanercept group); and 1 for each of use of prohibited concomitant medication (placebo), poor compliance (placebo), and early discontinuation of study drug (0.25% tanfanercept). The disposition of all study subjects is shown in Fig. 2. The demographics of all study subjects is shown in Table 1. The baseline disease characteristics in the study eye for all study subjects are shown in Table 2. Per the inclusion criteria, all subjects were 18 years or age or older. The mean age of all subjects was 42.6 ± 10.25 years; a majority of subjects (98%) were between 18 and 65 years of age and only 2 subjects (2%) were 65 years of age or older (both in the placebo treatment group). The gender disposition of all randomized subjects was 57% female and 43% male with similar representation of gender across the treatment groups. All subjects (100%) identified themselves as Asian.

**Table 1:** Demographics, all randomized subjects

	HBM9036 0.25% (*n* = 50)	Placebo (*n* = 50)	All subjects (*n* = 100)
*Age (years)*			
*n*	50	50	100
Mean (SD)	42.2 (9.77)	43.1 (10.80)	42.6 (10.25)
Age categories			
< 65 years	50 (100.0%)	48 (96.0%)	98 (98.0%)
≥ 65 years	0	2 (4.0%)	2 (2.0%)
< 35 years	14 (28.0%)	8 (16.0%)	22(22%)
≥ 35 years	36 (72.0%)	42 (84.0%)	78(78%)
Previous artificial tear use	3 (6.0%)	2 (4.0%)	5 (5%)
No previous artificial tear use	47 (94.0%)	48 (96.0%)	95(95%)
*Sex*			
Male	20 (40.0%)	23 (46.0%)	43 (43.0%)
Female	30 (60.0%)	27 (54.0%)	57 (57.0%)

*n* number of subjects, *OD* right eye, *OS* left eye, *SD* standard deviation

**Table 2:** Baseline disease characteristics (study eye)

	HBM9036 0.25% (*n* = 50)	Placebo (*n* = 50)
*Pre-CAE to post-CAE Ora Calibra*^®^* Ocular Discomfort scale *(0–4* scale, higher is worse*)		
Mean (SD)	0.6 (0.76)	0.6 (0.61)
*Pre-CAE to post-CAE inferior corneal staining *(0–4* scale, higher is worse*)		
Mean (SD)	1.27 (0.353)	1.25 (0.394)
*OSDI© *(0–100* scale, higher is worse*)		
Mean (SD)	53.12 (16.188)	51.98 (18.132)
*Best-corrected visual acuity *(*logMAR*)		
Mean (SD)	0.219 (0.1816)	0.234 (0.1504)
Pre-CAE TFBUT (seconds)		
Mean (SD)	3.63 (1.267)	3.76 (1.283)
*Post-CAE TFBUT* (*seconds*)		
Mean (SD)	3.75 (1.195)	3.53 (1.163)
*Unanesthetized Schirmer's test* (mm)		
Mean (SD)	3.6 (2.77)	3.5 (2.24)

*CAE* Controlled adverse environment, *logMar* logarithm of the minimum angle of resolution, *n* number of subjects, *OSDI*© Ocular surface disease Index©, *Q*^1^ first quartile, *Q*^3^ third quartile, *SD* standard deviation, *TFBUT* Tear-Film Break-Up Time

There were no notable differences in baseline characteristics between the 2 study groups. Both treatment groups had similar mean inferior corneal staining scores (ICSS) at all CAE measures (pre-CAE and post-CAE). ICSS pre-CAE challenge was 1.89 ± 0.395 for the active treatment group and 1.84 ± 0.479 for the placebo treatment group and ICSS post-CAE challenge was 3.15 ± 0.384 and 3.09 ± 0.522 for active and placebo, respectively. Importantly, no differences between treatment groups at baseline were observed for the primary endpoint measure, the change from pre-CAE to post-CAE ICSS (1.27 ± 0.353 and 1.25 ± 0.394 for active and placebo, respectively).

### Efficacy of tanfanercept

#### Sign assessments of DED

**Fig. 3:**
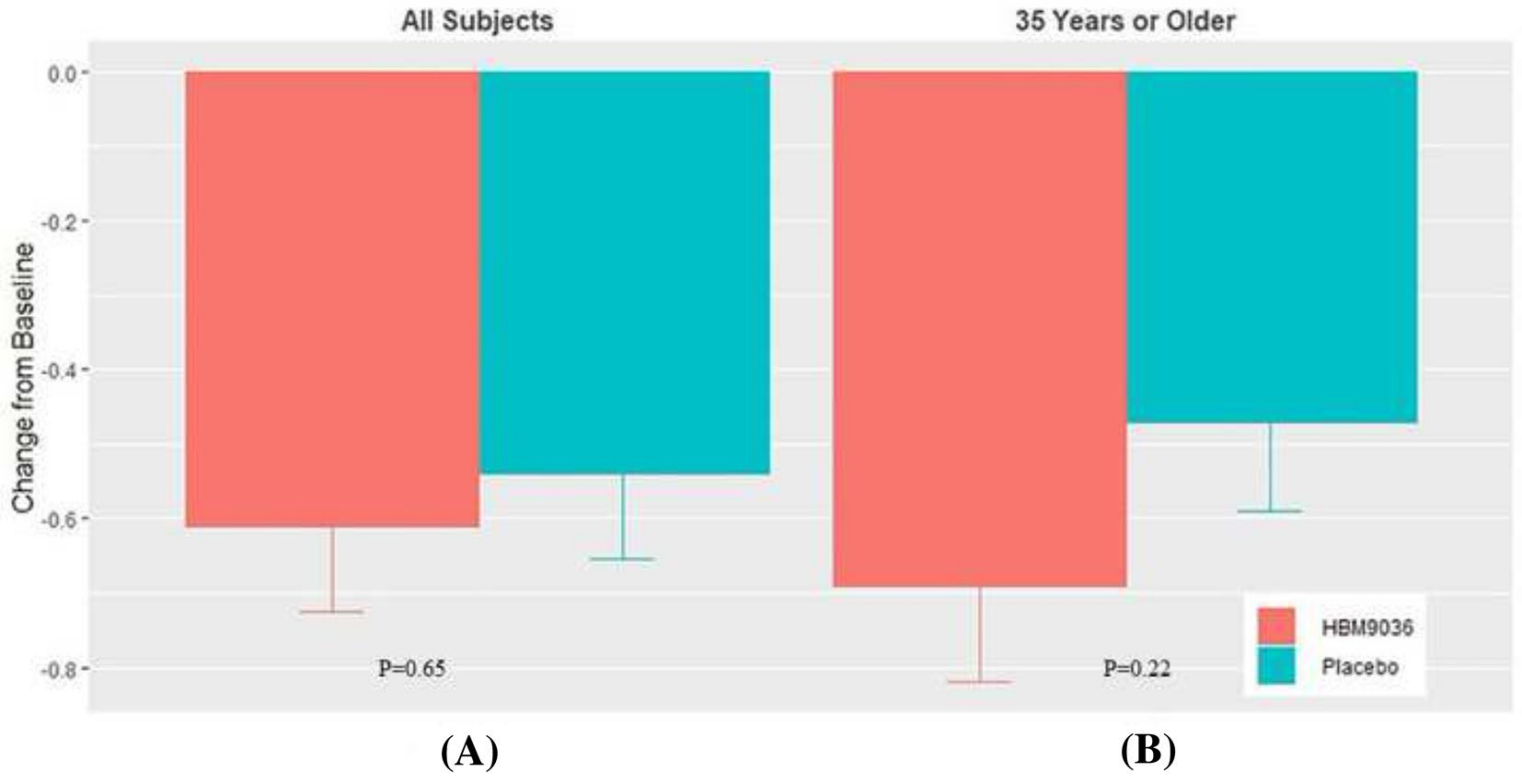
Change from baseline in pre- to post-CAE ICSS at Visit 6 (Day 57). All subjects in the tanfanercept treatment group showed clinical improvement in change from baseline ICSS when compared to subjects in the placebo treatment group. In a subset of the subjects in the study age 35 years or older, clinical improvements in change from baseline ICSS were further increased compared to placebo

**Fig. 4:**
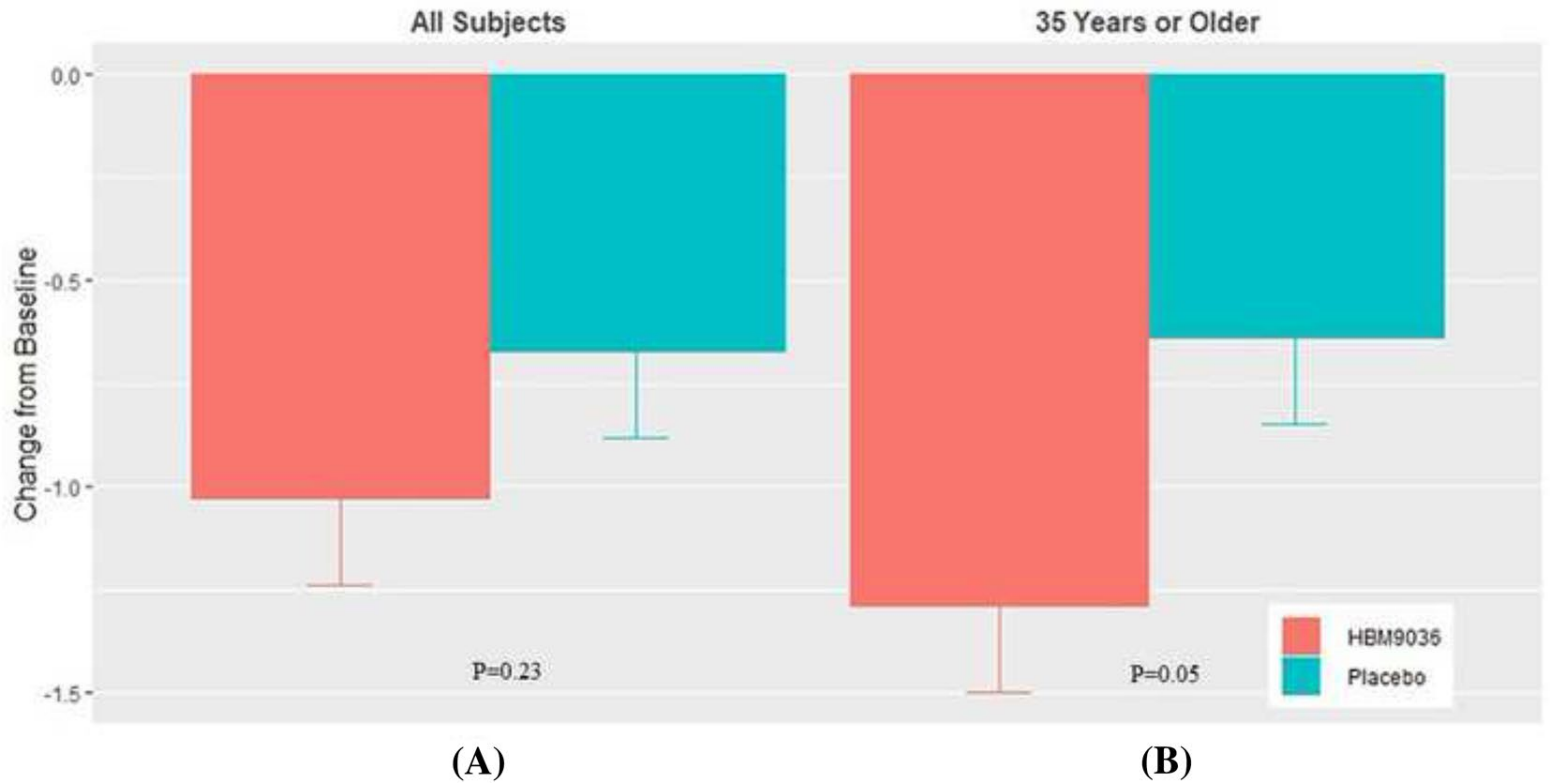
Change from baseline in pre- to post-CAE TCSS at Visit 6 (Day 57). All subjects in the tanfanercept treatment group showed clinical improvement in change from baseline TCSS when compared to subjects in the placebo treatment group. In a subset of the subjects in the study age 35 years or older, clinical improvements in change from baseline TCSS were further increased compared to placebo

Overall, the 0.25% tanfanercept treatment group showed clinical improvements from baseline in the sign assessments of DED, specifically in the change in ICSS and TCSS from pre- to post-CAE challenge measures, unanesthetized Schirmer’s test, and TFBUT compared to the placebo group. The change from baseline ICSS pre- to post-CAE challenge for the 0.25% tanfanercept group compared to placebo was − 0.61 ± 0.11 and − 0.54 ± 0.11 (LS mean ± SE) for active and placebo, respectively) (Fig. 3A). Notably, the magnitude of the observed clinical improvements in ICSS was greater in subjects aged 35 years or older (Fig. 3B). Improvements were not limited to the inferior corneal region as similar results favoring active treatment over placebo were observed for change from baseline total corneal staining scores (TCSS) pre- to post-CAE challenge [− 1.03 ± 0.21 and − 0.67 ± 0.21 (LS mean ± SE), respectively] (Fig. 4A). As observed with TCSS, benefits (− 1.29 ± 0.24 (LS mean ± SE), CI (− 1.7064, − 0.8196) over placebo (− 0.64 ± 0.22 (LS mean ± SE), CI (− 1.0712, − 0.2088)] treatment were of greater magnitude in the subgroup of the population age 35 years and older (Fig. 4B).

**Fig. 5:**
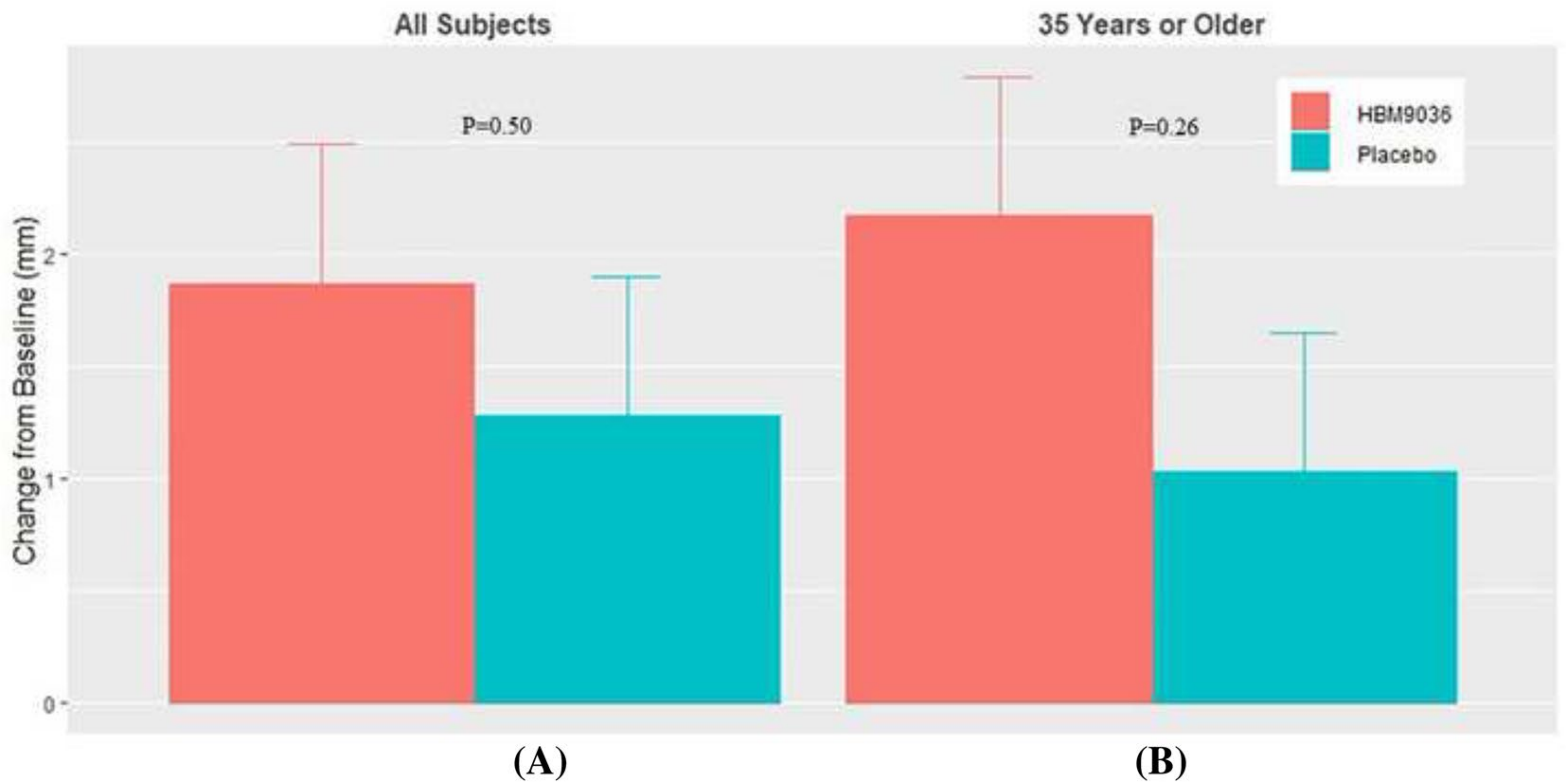
Change from baseline in Schirmer’s score at Visit 6 (Day 57). All subjects in the tanfanercept treatment group showed clinical improvement in change from baseline Schirmer’s score when compared to subjects in the placebo treatment group. In a subset of the subjects in the study age 35 years or older, clinical improvements in change from baseline Schirmer’s score were further increased compared to placebo

In addition, an efficacy signal of increased tear production and quality was observed via Schirmer’s test and TFBUT at Visit 6 (Day 57). Improvements in Schirmer’s test scores were demonstrated in favor of active treatment [1.87 ± 0.62 (LS mean ± SE)] when compared to placebo [1.28 ± 0.62 (LS mean ± SE)] (Fig. 5A), and a similar result was observed for improvements in mean TFBUT (means difference (active–placebo) = 1.21 s, *p* = 0.4491). The trend of greater benefit in the subgroup of the study population aged 35 years and older was maintained for tear production and quality with the magnitude of the improvement over baseline favoring treatment with 0.25% tanfanercept [2.17 ± 0.74 (LS mean ± SE), CI (0.7196, 3.6204)] over placebo [1.03 ± 0.66 (LS mean ± SE), CI (− 0.2636, 2.3236)] (Fig. 5B).

#### Symptom assessments of DED

**Fig. 6:**
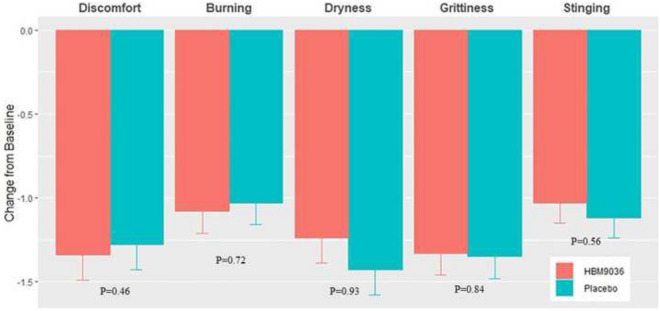
Ora Calibra^®^ Ocular Discomfort and 4-Symptom Questionnaire Score at Visit 6 (Day 57). Subjects answered the Ora Calibra^®^ Ocular Discomfort and 4-Symptom Questionnaire at each office visit throughout the study. The questionnaire measures ocular discomfort and 4 symptoms of DED: burning, dryness, grittiness, and stinging. There was no consistent improvement in dry eye symptoms for the 0.25% tanfanercept treatment group over placebo treatment group compared to baseline symptom scores measured at Visit 2

Substantial improvements from baseline were demonstrated in the 0.25% tanfanercept group for all symptoms of DED assessed. However, improvement from baseline was also observed in the placebo group. For most symptoms assessed by the Ora Calibra^®^ 4-symptom questionnaire, no consistent improvements were demonstrated for treatment with 0.25% tanfanercept over placebo (Fig. 6).

### Safety and tolerability of tanfanercept

Tanfanercept is generally safe and well tolerated with similar comfort level with placebo and demonstrated improvements in the signs of DED compared to placebo (Table 3). There were an equal number of treatment-emergent adverse events (TEAEs) in both active and placebo treatment arms (13 subjects, 26.0%). Similarly, non-ocular TEAEs were approximately equal between groups (9 subjects (18.0%) and 10 subjects (20.0%), active and placebo, respectively; however, ocular AEs were more prevalent in the subjects treated with 0.25% tanfanercept (7 subjects, 14.0%) as compared with placebo (4 subjects, 8%). Of the ocular AEs experienced by subjects during the study, the most frequently reported were conjunctivitis and conjunctival redness [3 subjects (6.0%) in the tanfanercept group and 0 subjects (0.0%) in the placebo group, each]. All other ocular AEs were infrequent reported by less than 3 subjects in either treatment group. All AEs reported during the study were mild, with the exception of one moderate AE (not related) reported in the 0.25% tanfanercept treatment group. No significant findings were found in any of the safety measures assessed during the study.

**Table 3:** Safety and tolerability of treatment with 0.25% tanfanercept

	0.25% tanfanercept	Placebo
	(*n* = 50)	(*n* = 50)
*Safety, n* (%)		
Number of Subjects with Treatment-*emergent AE*	13 (26.0%)	13 (26.0%)
Number of subjects with ocular AE	7 (14.0%)	4 (8.0%)
Number of subjects with non-ocular TEAE	9 (18.0%)	10 (20.0%)
Number of subjects with SAE	0 (0.0%)	0 (0.0%)
*Most frequent ocular AE*		
Conjunctivitis	3 (6.0%)	0 (0.0%)
Conjunctival redness	3 (6.0%)	0 (0.0%)
*Tolerability*		
Drop comfort scale, mean (SD)		
Upon installation	3.7 (2.26)	3.8 (1.98)
*p*-value, *t*-test	0.7074	–
1 min post-instillation	3.4 (2.18)	3.5 (2.12)
*p*-value, *t*-test	0.7458	–
2 min post-installation	3.1 (2.20)	3.5 (2.10)
*p*-value, *t*-test	0.3796	–

*AE* adverse event, *n* number of subjects, *SAE* serious adverse event, *SD* standard deviation

Drop comfort was assessed with the Ora Calibra^®^ Drop Comfort Scale at baseline (Visit 2) in the study eyes of the safety set of all patients who were randomized into either arm of the study. Treatment with 0.25% tanfanercept was generally well tolerated with a similar comfort level to treatment with placebo. No significant differences [*p* = 0.7074 (*t*-test)] in drop comfort between the two groups upon instillation of the study drug were reported (3.7 and 3.8, active and placebo, respectively) (Table 1). Similarly, no significant reported differences in drop comfort continued through observations at 1 or 2 min post-instillation [3.4 ± 2.18 and 3.1 ± 2.20, active, and 3.5 ± 2.12 and 3.5 ± 2.10, placebo, respectively; *p* (1 min) = 0.7458 and *p* (2 min) = 0.3796 (t-test)]. Overall, these data support a safe and comfortable profile for HBM9036 as a topical treatment for dry eye.

## Discussion

The data presented here establish the clinical safety and efficacy of 0.25% tanfanercept ophthalmic solution as a novel treatment for DED in Chinese adult patients with moderate-to-severe DED. This study employed the first-in-China use of the Ora CAE to screen for and exacerbate responses in the signs and symptoms of DED in a controlled manner.

In this phase 2 study, tanfanercept demonstrated efficacy in the signs and symptoms of dry eye induced by the CAE as compared to placebo. The 0.25% tanfanercept treatment group showed improvement in inferior CSS pre- to post-CAE change from baseline scores when compared to placebo (− 0.61 and − 0.54, respectively). Improvements in fluorescein staining score were not limited to the inferior ocular region with TCSS pre- to post-CAE challenge change from baseline scores also demonstrating benefit of treatment with active when compared to placebo − 1.03 and − 0.67, respectively). Treatment with 0.25% tanfanercept mediated positive effects on tear production and quality, reflected in Schirmer’s score improvement demonstrated in favor of active (1.87) as compared to placebo (1.28) and a mean difference in TFBUT of 1.21 favoring tanfanercept treatment. Ultimately, improvements in dry eye signs from baseline for those treated with 0.25% tanfanercept compared to placebo were evident across all subjects completing the study with no major protocol deviations (PPS). In addition to observation of improvements in DED signs in the 0.25% tanfanercept treatment group, there were demonstrated improvements in DED symptoms as measured by the Ora Calibra^®^ Ocular Discomfort and 4-Symptom Questionnaire at the secondary analysis endpoint (Visit 6, Day 57) for all subjects in the study. It is notable that there was a slight difference in ocular discomfort and burning favoring treatment with tanfanercept over placebo. While this could result in clinically important reduction of DED symptoms for the subject, the statistical significance of this treatment-mediated improvement was not established.

It is noted that the age distribution in this study was relatively younger than studies previously reported including the Diquas^®^ phase 3 trial in China and pivotal studies for other FDA approved anti-inflammation ophthalmics [7, 8, 20, 21]. Age is recognized as a major internal factor driving development of DED as such DED is a disease more prevalent and severe in older populations [4]. The contrarily younger population studied here could be considered a limitation resulting from the study’s relatively small sample size and single-center design. Given that most DED studies are conducted with older populations more reflective of DED distribution within the global population, a subgroup of age >  = 35 was analyzed herein to ameliorate the limitation of this study population. In the subgroup of the PPS, study subjects age 35 years and older comprising approximately 78% of study subjects, improvements of signs were further pronounced. Overall, the effect sizes in ICSS, TCSS and Schirmer’s score were greater (− 0.69 ± 0.13 vs. − 0.47 ± 0.12, − 1.29 ± 0.24 vs. − 0.64 ± 0.22, and 2.17 ± 0.74 vs. 1.03 ± 0.66 [active vs. placebo], respectively). For subjects treated with 0.25% tanfanercept, greater magnitudes of improvement from baseline and as a result greater differences between active treatment and placebo were consistently noted for the signs of DED. As seen in this study, the older population subgroup showed a larger effect size than overall younger population. In consideration of the multi-factorial etiology of DED, disease in younger populations may be increasingly driven by exogenous factors. Thus, this population may be more prone to have spontaneous improvements upon external environment changes making them an unstable and less representative population for DED trials. Despite this, younger populations can still attain similar disease severity as older populations at some time point.

Since all subjects in the trial showed similar marked improvement from baseline across the majority of the assessed DED symptoms regardless of treatment (active or placebo) received, it can be concluded that there was a strong placebo effect experienced by the subjects in this study. It is possible that the placebo used in this study, vehicle that is equivalent to active drug minus the active component (0.25% tanfanercept), closely resembled tear substitutes and therefore could have provided temporary symptom improvement in the severity of DED symptoms experienced. A strong placebo effect would have resulted in a masking of any 0.25% tanfanercept-mediated improvements in symptom severity over placebo treatment. The results of this study further demonstrate the difficulty of observing efficacy signals in both DED sign and symptom in the same population.

This study was designed to compare the safety & efficacy of tanfanercept (HBM9036) to a previously conducted phase 2 study (VELOS-1) in the USA where statistically significant improvements compared to placebo before and after exposure to the dry environment. The results from this phase 2 study conducted in China replicate the efficacy in sign improvements observed in the VELOS-1 study and are most apparent at the primary efficacy endpoint of fluorescein ICSS pre- to post-CAE change from baseline. At this endpoint, the change from baseline was near equivalent to that observed in the USA study (*Δ* = 0.22 in China, *Δ* = 0.25 in the USA [> 35 age group]). A key difference between the studies is that there was a markedly younger study population in China in comparison with the population in the USA study highlighting the importance of evaluating the efficacy within this study among the > 35 age subgroup. For the symptoms observed to therapy within this study, a placebo effect is a possible explanation. This effect is commonly seen in numerous trials that evaluate symptoms [22]. Additionally, this effect was more apparent in subjects who have not used artificial tears (US P2). The proportion of subjects in this study who have used artificial tears was much lower (*n* = 5, 5%) compared to subjects in both, the US study and other studies [7, 23]. Despite the key differences in subject populations between the two studies (USA and China), the replication of clinically significant efficacy at the primary endpoint signals the robust potential of 0.25% tanfanercept to be highly efficacious for the treatment of DED. In addition to demonstrating efficacy in signs of DED, treatment with 0.25% tanfanercept had a favorable safety profile in this study. No SAEs were reported. All AEs, but one, were classified as mild in severity, and all AEs were resolved by the end of the study. In addition, similar drop comfort levels within a comfortable range for subjects were observed for treatment with active and placebo. Ultimately, treatment with 0.25% can be deemed comfortable and well tolerated without any new safety risks.

## Conclusion

These results support that treatment with 0.25% tanfanercept is an exciting innovation, and therapeutic candidate, for the treatment of DED as an alternative to the current standard of care therapeutics. Similarly, the previous non-clinical and clinical studies of tanfanercept suggest alleviation of the bioavailability and ocular tissue penetration challenges faced by biologic anti-inflammatory therapeutics. Overall, this study presents the strong, favorable safety profiles and improvement tendency in DED sign and symptom improvements demonstrated by treatment with 0.25% tanfanercept that support its continued development through phase 3 studies in China.

## Data Availability

The datasets used and/or analyzed during the current study are available from the corresponding author on reasonable request.

## Abbreviations

BID: Two times daily
CAE: Controlled adverse environment
DED: Dry eye disease
FAS: Full analysis set
ITT: Intent-to-treat
CSS: Corneal staining score
ICSS: Inferior corneal staining score
TCSS: Total corneal staining score
ODS: Ocular discomfort scale
OSDI: Ocular surface disease index
PPS: Per-protocol set
MedDRA: Medical dictionary for regulatory activities
SD: Standard deviation
SS: Safety set
AE: Adverse event
TFBUT: Tear-film break-up time
TNF: Tumor necrosis factor
TNFR: TNF receptor
WHO: World health organization
